# Linc00423 as a tumor suppressor in retroperitoneal liposarcoma via activing MAPK signaling pathway through destabilizing of NFATC3

**DOI:** 10.1038/s41419-019-1658-2

**Published:** 2019-06-03

**Authors:** Yong Zhang, Hanxing Tong, Junyi He, Yebo Shao, Xi Guo, Rongyuan Zhuang, Jue Yang, Ju Liu, Yuqin Ding, Wenshuai Liu, Weiqi Lu, Yuhong Zhou

**Affiliations:** 1 Department of General Surgery, Zhongshan Hospital, Fudan University, Shanghai, 200032 China; 2 Department of General Surgery, Xiamen Branch, Zhongshan Hospital, Fudan University, Xiamen, 361015 China; 3 Department of Oncology, Zhongshan Hospital, Fudan University, Shanghai, 200032 China; 4 Department of Vascular Surgery, Zhongshan Hospital, Fudan University, Shanghai, 200032 China; 5 Department of Pathology, Zhongshan Hospital, Fudan University, Shanghai, 200032 China; 6 Department of Radiology, Zhongshan Hospital, Fudan University, Shanghai, 200032 China

**Keywords:** Cancer genomics, Sarcoma

## Abstract

Unraveling the noncoding RNA expression networks governing cancer initiation and development is essential while remains largely uncompleted in retroperitoneal liposarcoma (RLS). Through RNA-seq technologies and computational biology, deregulated long noncoding RNAs (lncRNAs) are being identified and reveal that lncRNAs are implicated in serial steps of RLS development. High-throughput sequencing with computational methods for assembling the transcriptome of five paired RLS patient’s tissues. We found that long intergenic noncoding RNA 423 (linc00423) was downregulated in RLS tissues. Gain-of-function assays revealed that overexpressed linc00423 obviously inhibited RLS cell growth in vitro and in vivo. Additionally, RNA sequence, RNA-pulldown and RIP assays evidenced that linc00423 involved in MAPK signaling pathway via destabilizing of nuclear factor of activated T-cells 3 (NFATC3). Summing up, our findings demonstrated that linc00423 acted as the tumor suppressor in RLS cells through regulating the protein level of NFATC3 at a post-transcriptional level and negatively regulated the MAPK signaling pathway at a transcriptional level. Linc00423 might serve as a candidate prognostic biomarker and a target for novel therapies of RLS patients.

## Introduction

Soft tissue sarcoma (STS) is the rare mesenchymal tumors mostly arising from embryonic mesoderm of soft tissue that can differ significantly in their disease presentation. As the most common primary malignancy of soft tissues, retroperitoneal liposarcoma (RLS) was located in the retroperitoneum and exhibited different aggressive potentials, reflecting their morphologic diversity^1–3^. Until now, due to lack of characteristic clinical presentations, a traditional diagnosis system, which involves surgery, chemotherapy (CT), and radiotherapy (RT) of RLS, also meets considerable challenges with the poor prognosis of patients^4–6^. Genetic alterations take up the main predisposing factors of this disease, and studies on RLS molecular pathways and genetic mutations have identified new treatment targets with promising results^7^.

Investigations over the past few decades have principally focused on how encoded proteins regulate myriad levels of gene transcription. Recently, benefit by high-resolution microarray and genome-wide sequencing technology, control of gene expression by noncoding RNAs has raised great concern^8^. Rather than “transcriptional noise”, these non-protein-coding RNAs are of functional significance^9–11^. Based on the transcript size of noncoding RNAs, they can grouped into two major classes: small noncoding RNAs (<200 nt) and long noncoding RNAs (lncRNAs; ≥ 200 nt). lncRNAs initially were considered as byproduct of transcription of RNA polymerase II, are mostly spliced, 5′ capped, and polyadenylated at 3′ end^12–14^. Although only a few of the thousands of noncoding RNAs have been analyzed experimentally, many involved in various physiological and pathological processes at epigenetic, transcriptional, or post-transcriptional level to regulate the expression of related genes which links to human disease including various cancers, and various disease^15–20^. However, an enormous number of lincRNAs remains to be elucidated and characterized.

In this study, we screened a candidate tumor-suppressor lincRNA, linc00423, from five paired RLS and tumor-adjacent nontumor tissues via the high-throughput sequencing with computational methods for assembling the transcriptome. Our results showed that linc00423 has been downregulated in RLS tissues with genomic copy number deletion, and overexpression of linc00423 could inhibited cell growth and colonies formation in vitro and in vivo. For mechanistic investigations revealed that linc00423 can control RLS progression by direct binding with and regulated the stability of the nuclear factor of activated T-cells 3 (NFATC3). Taken together, these results suggest that linc00423 may be as the promising molecular target for RLS therapy.

To our knowledge, rare study has reported the functional relevance of lncRNA-regulated mechanisms in the cancer preventing efficacy of RLS. Our findings will provide new insights into the molecular functions of linc00423 as well as its regulatory mechanisms in RLS.

## Materials and methods

### Patients and tissue samples

A total of 42 paired and 150 paired frozen RLS tissues and normal human enterocoelia tissue were randomly obtained with informed consent from patients who underwent radical resections in the Zhongshan Hospital affiliated with the Fudan University (Shanghai, China) between 2014 and 2017. All patients without CT or RT before surgery. This study was approved by the Institutional Review Board of Fudan University, with all of the participants signed an informed consent form.

### Cell culture

HEK293T, 93T449, and 94T778 cells were cultured at 37 °C in an atmosphere containing 5% CO_2_ and in Dulbecco’s modified Eagle’s medium (Invitrogen, USA) supplemented with 10% fetal bovine serum and penicillin-streptoMycin (Gibco, USA). Where indicated, cells were treated with 50 μg/ml cycloheximide (CHX) (Selleck, USA) for 24 h with serum-free DMEM. All cells were routinely tested as mycoplasma free with the kit from Biotool (B39032).

### RNA extraction and real-time qPCR analysis

Total RNA was isolated using Trizol reagent (Invitrogen). First-strand cDNA was generated using the PrimeScript™ Reverse Transcriptase Kit (Takara, Dalian, China) according to the manufacturer’s protocol. Real-time qPCR was performed in the 7900HT Fast Real-Time PCR System (Applied Biosystems, USA) using SYBR Green (Takara). GAPDH was employed as an endogenous control for mRNAs and lncRNAs. For linc00423 qRT-PCR, the primer pair 5′-AGTCAAGGCCCCCAGGTATCT-3′ (forward) and 5′-GCTGGGATTATTCTGATAGGGTC-3′ (reverse) was used to amplify a 213-bp product. Human GAPDH was using primers 5′-GAGAGAGAGCCTGGACCTCA-3′ (forward) and 5′-TGGGTCCTCTAGCAGTCTCC-3′ (reverse) as the loading control. The relative expression of RNAs was calculated using the comparative Ct method.

### Vector construction, small interfering RNA (siRNA) synthesis and transfection

The cDNA encoding the CDS of NFATC3 and full length of linc00423 was PCR-amplified by Thermo Scientific Phusion Flash High-Fidelity PCR Master Mix (Thermo) and subcloned into the EcoRI and XhoI sites, BamHI and EcoRV sites of the pcDNA3.0 vector (Invitrogen) respectively, named NFATC3 and linc00423. The siRNAs specifically targeting linc00423 and NFACT3, and control siRNA were synthesized by GenePharma (Shanghai). Cells were transfected with the plasmids or siRNAs using Lipofectamine 2000/3000 (Invitrogen) according to the manufacturer’s protocol.

### Proliferation and colony formation assay

For cell proliferation assays, a total of 1500 cells were seeded into 96-well plates. After 1, 3, and 5 days of culture, cell proliferation was assessed using the Cell Counting Kit-8 (Dojindo Laboratories, Janpan) according to the manufacturer’s protocol. The cell proliferation curves were plotted using the absorbance at each time point. For colony formation assays, 1500 cells were seeded in the 6-well plates and incubated with normal medium for 10–14 days. Clones were fixed and stained with 0.5% crystal violet, and the number of colonies was counted.

### Immunofluorescence

Cells seeded on glass coverslips in 3.5 cm dish, which were fixed in 4% absolute ethyl alcohol and permeabilized with 0.5% TritonX-100/PBS. Cells were blocked with 2.5% BSA-PBS overnight in 4 °C, and incubated with primary antibody for 3 h at room temperature, followed by incubation with fluorescent-dye conjugated secondary antibody for 1 h, and then stained with DAPI.

### Flow cytometric analysis of cell cycle distribution

Cells were harvest after 48 h transfection and washed with ice-cold PBS, and fixed with 75% ethanol overnight at −20 °C. Fixed cells were rehydrated in PBS for 10 min and incubated in RNase A for 30 min at 37 °C, then cells were subjected to PI staining followed by flow cytometric analysis using an FACScan instrument (BD Biosciences, USA).

### RNA sequencing analysis and gene set enrichment analysis (GSEA)

The RNAs from 93T449 cells stably overexpressing linc00423 or control were isolated, quantified and purified prior to generation of the cDNA library as the manufacturer’s instructions. The sequencing libraries were generated using the NEX flex Rapid RNA-seq Kit (Bioo scientific, USA) from Illumina according to the manufacturer’s instructions. We used GSEA v2.2.0 to perform GSEA on various gene signatures. Gene sets were obtained from published gene signatures. Statistical significance was assessed by comparing the enrichment score to enrichment results generated from 1000 random permutations of the gene set to obtain FDR *q* values.

### RNA pulldown assay and RNA immunoprecipitation (RIP) assay

Linc00423-sense and linc00423-antisense were in vitro-transcribed, respectively, from the vector pcDNA3.0-linc00423 and biotin-labeled with the Biotin RNA Labeling Mix (Roche, USA) and T7 RNA polymerase (Roche, USA). The in vitro-transcribed transcripts were treated with RNase-free DNase I (Takara, Japan), and purified with an RNeasy Mini Kit (Qiagen, Germany). One microgram of whole-cell lysates from 93T449 cells was incubated with 3 mg of purified biotinylated transcripts for overnight at 4 °C; RNA and protein complexes were isolated with streptavidin agarose beads (Invitrogen, USA). After incubation overnight, use 500 mM, 750 mM, and 1 M NaCl for washing the complex. Elutes were analyzed by liquid mass spectrometry.

For the anti-NFATC3 RIP assay, we use the Magna RIP RNA-Binding Protein Immunoprecipitation Kit (Millipore) according to the manufacturer’s instructions. 93T449 cells were transfected with linc00423-sense and linc00423-antisense expressing plasmids. After 48 h, cells were used to perform RIP experiments using an NFATC3 antibody (sc-23814, Santa cruz biotechnology, USA).

### 5′ and 3′ rapid amplification of cDNA ends (RACE) analysis, subcellular fractionation analysis

We use the SMART™ RACE cDNA Amplification Kit (Takara, Japan) to amplification the 3′ and 5′ end of linc00423 according to the manufacturer’s protocol. Subcellular fractionation analysis were performed use the Nuclear/Cytosol Fractionation Kit (Pierce, USA).

### Xenograft transplantation assay

Approximately 5.0 × 10^6^ 93T449 cells were suspended in 50 μl of PBS and stably transfected with pcDNA3.0-linc00423, or vector controls were injected subcutaneously into the right side of the posterior flank of female BALB/c athymic nude mice (Department of Medicine, Fudan University) at 5–6 weeks of age. Tumor growth was examined every other day with a vernier caliper. Tumor volumes were calculated by using the equation: *V* = *A* × *B*^2^/2 (mm^3^), where A is the largest diameter and B is the perpendicular diameter. After 5 weeks, all mice were killed and necropsies were carried out.

### Statistical analysis

The Graphpad Prism7.0 statistical analysis software was used for the statistical analysis of the experimental data. The significance of differences between groups was estimated by Student’s *t*-test. Multiple group comparisons were analyzed with one-way ANOVA. A *P* value less than 0.05 was considered significant.

## Results

### Linc00423 is downregulated in human RLS tissues with copy number deletion

**Fig. 1:**
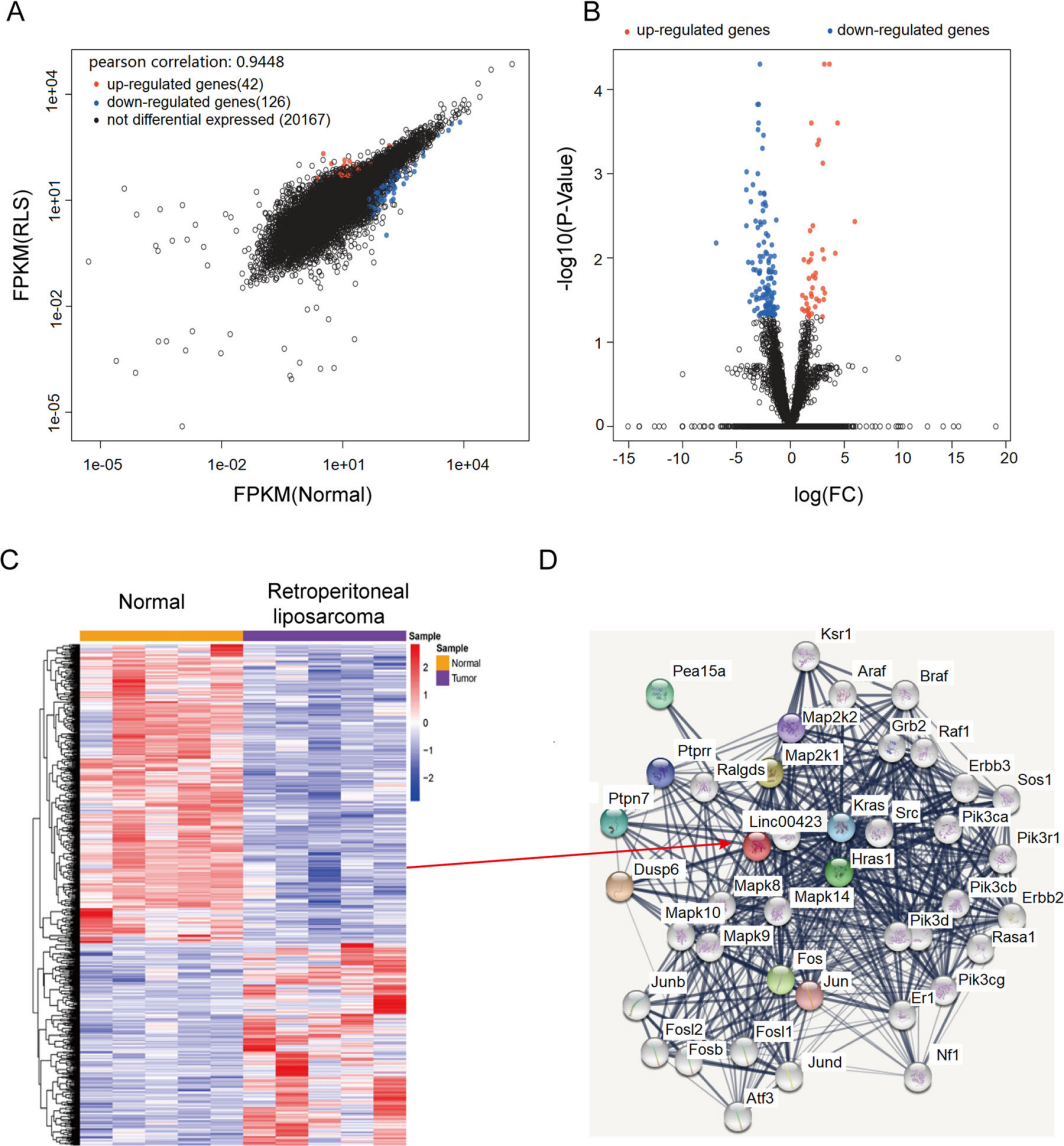
Screened linc00423 in five paired RLS tissues by RNA sequencing. **a**, **b** Scatter plot (**a**) and volcano plot (**b**) results of reliability of RNA sequence. **c** Heat map showed clustering analysis of RNA sequencing by five-paired RLS tissues and normal tissues. Each row represents a gene and each column represents a tissue samples. Red represents upregulated genes and blue represents downregulated genes. **d** Bioinformatics analysis of the networks of key candidate long noncoding RNAs in the development and progression of RLS

To explore the oncogenes and tumor suppressor genes in RLS, we first performed RNA sequencing of five paired RLS and tumor-adjacent nontumor tissues. Through clustering analysis of the raw data, we aimed to find the tumor suppressor genes in RLS (Fig. 1a–c). To further determine which lncRNA among these 126 novel tumor suppressors, we next examined the potential driver roles of the candidates by combining with a public database to analyze the interaction network of known genes or signaling pathways (Fig. 1d). A set of 10 potential genes in aberrant genes were chosen for further study (Supplementary Fig. 1A). Specifically, based on the expression level and biological functions, Linc00423 was selected according to the results of proteins network analysis.

**Fig. 2:**
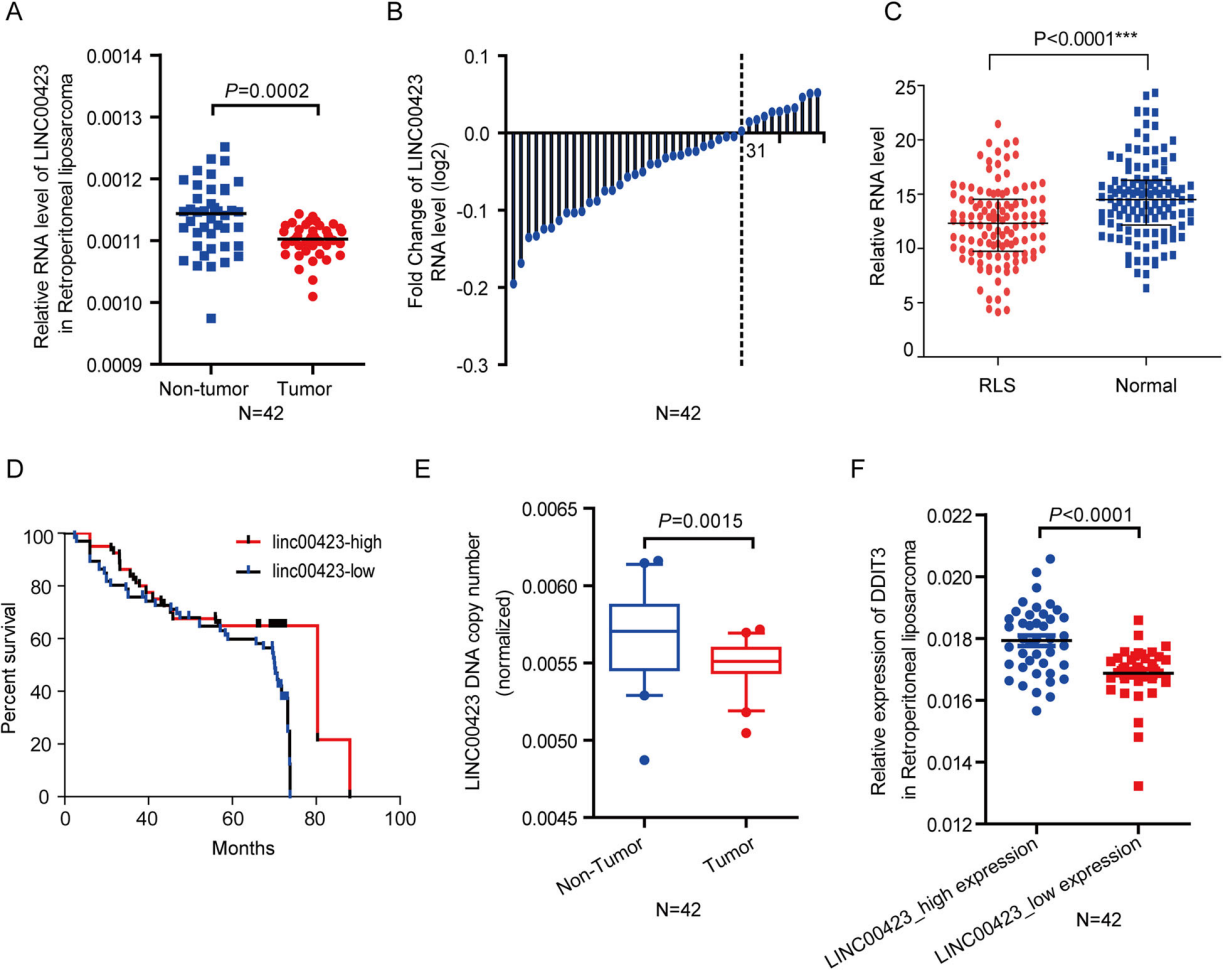
linc00423 low-expressed in RLS due to DNA copy number deletion. **a** QRT-PCR results showed that linc00423 expression was significantly downregulated in 42 pairs of RLS tissues and nontumor tissues (NTs). **b** Fold change of linc00423 in 42 paired of RLS tissues and NTs. **c** The expression profile of linc00423 in 150 paired RLS and paired normal tissues. **d** Kaplan–Meier curves for prognistic value for linc00423 in 150 paired RLS corhort. **e** linc00423 with the copy number deletions in 42 paired RLS tissues and NTs DNA samples were determined by qRT-PCR. **f** linc00423 expression level was positively correlated with the DDIT3 in 42 paired RLS tissues

First, the RNA levels of linc00423 were confirmed by quantitative real-time polymerase chain reaction (qPCR) analysis in 42 paired RLS tissues and paired tumor-adjacent nontumor tissues. Compared with matched normal tissues, linc00423 was significantly downregulated in RLS tissues (Fig. 2a, *P* = 0.0002). Among 42 paired tissues, up to 76% of samples with linc00423 downregulated (Fig. 2b). Besides, we expanded the cohort size for testing the RNA level and overall survival of linc00423 in 150 paired RLS tissues. Results showed linc00423 has the significantly lower RNA level contrast with normal tissues (Fig. 2c). The univariate Cox proportional hazards regression method revealed that gain of linc00423 showed the prognostic value in RLS tissue with *P* value less than 0.05 (Fig. 2d). TCGA data also confirmed the expression profile of linc00423 in 408 paired RLS tissues and normal tissues (Supplementary Fig. 1B, C). To wonder the low-expressed mechanism of linc00423, we examined the copy number alterations of linc00423 in matched 42 paired DNA sample of RLS tissues. The results revealed linc00423 with a significantly copy number deletion in RLS tissues (Fig. 2e, *P* = 0.0015). DNA damage inducible transcript 3 (DDIT3) is a member of the enhancer-binding protein (C/EBP) family of transcription factors^21^. DDIT3 was a dominant-negative inhibitor to preventing their DNA binding activity, via forming heterodimers with other C/EBP members^22,23^. So, DDIT3 is usually known as the negative molecule marker of the malignant proliferation of tumor cells. Therefore, we also analyze that the correlation of linc00423 and DDIT3 results showed that linc00423 had a positive correlation with DDIT3, suggesting that linc00423 may be a tumor-suppressor gene in RLS (Fig. 2f, *P* < 0.0001). These data together indicated that linc00423 was significantly downregulated and related to the overall survival in RLS tissues, and might be involved in the progression of RLS.

### Overexpressed linc00423 inhibited cell proliferation in vitro and in vivo

**Fig. 3:**
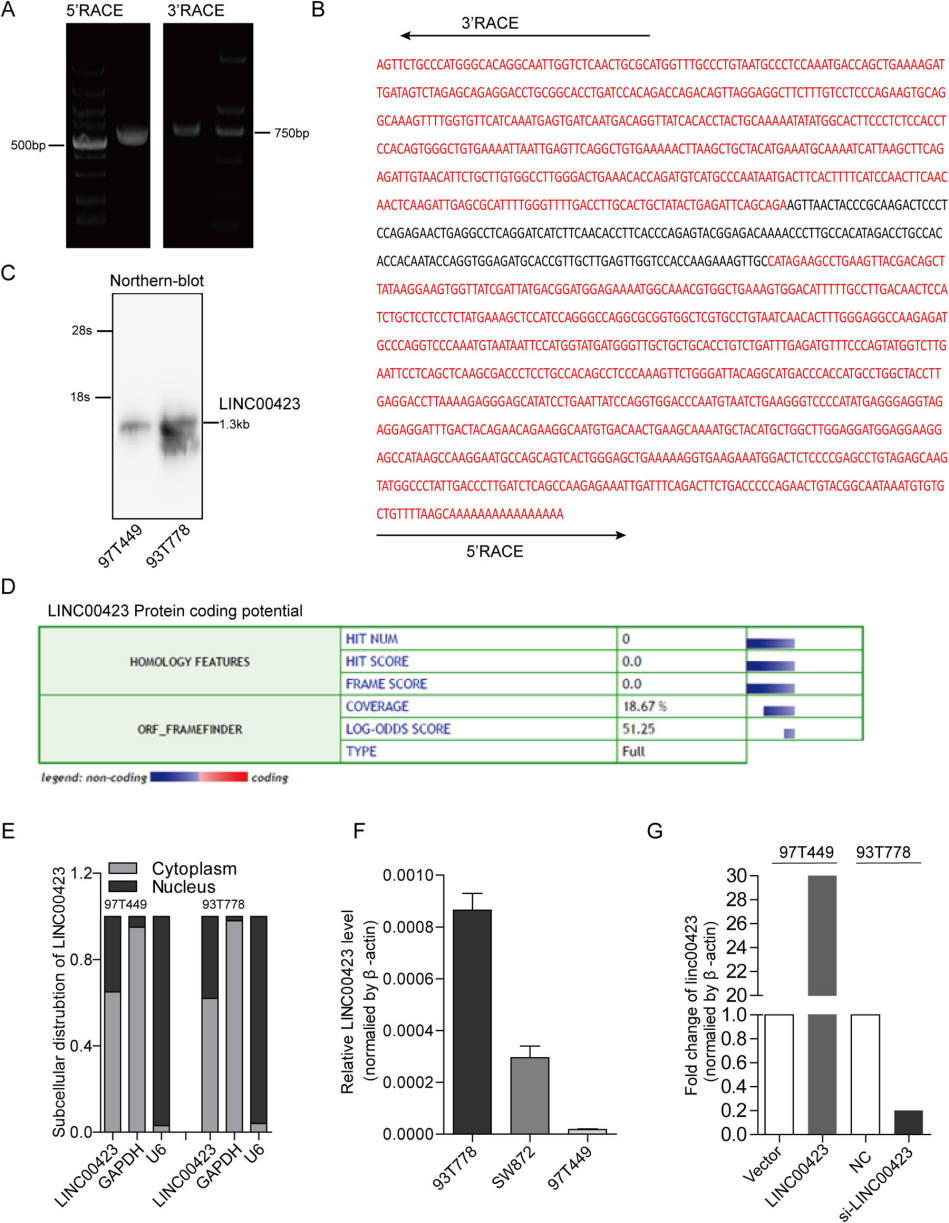
Basic feature of linc00423 in RLS cell lines. **a** Representative image of PCR products from the 3′ to 5′ RACE of linc00423. **b** The full sequence of linc00423 in 97T449 cells, red-highlight sequence present the sequencing results of 3′ and 5′ RACE. **c** Northern-blot verified the full-length of linc00423 in 97T449 and 93T778 cell lines. **d** Predicted the protein-coding potential of linc00423 by Coding Potential Calculator. **e** RNA level of lin00423 in cytoplasmic and nuclear extract of RLS cells. U6 as the internal control of nuclear, GAPDH as the internal control of cytoplasmic. **f** The background expression level of linc00423 in three RLS cell lines. **g** Build the stable overexpressed cell lines of linc00423 in 97T449 cells and verified the effective siRNAs in 93T778 cells

We first analyze the basic characteristic of linc00423 in RLS cells. We performed the RACE assay (rapid amplification of cDNA ends) and northern-blot assay to confirm the Linc00423 is a ~1.3-kb-long intergenic nonprotein-coding RNA in 97T449 and 93T778 cells (Fig. 3a–c). By Coding Potential Calculator (http://cpc.cbi.pku.edu.cn/), linc00423 has low protein-coding ability (Fig. 3d). Next, we examined the subcellular localization of linc00423, finding that linc00423 predominately resides in the cytoplasm in 97T449 and 93T778 cells (Fig. 3e). linc00423 has the significantly different expression level in RLS cell lines; we choose the 93T778 cell line that expresses a relatively high level of linc00423 and the 97T449 cell line that expresses a relatively low level of linc00423 for further study (Fig. 3f).

**Fig. 4:**
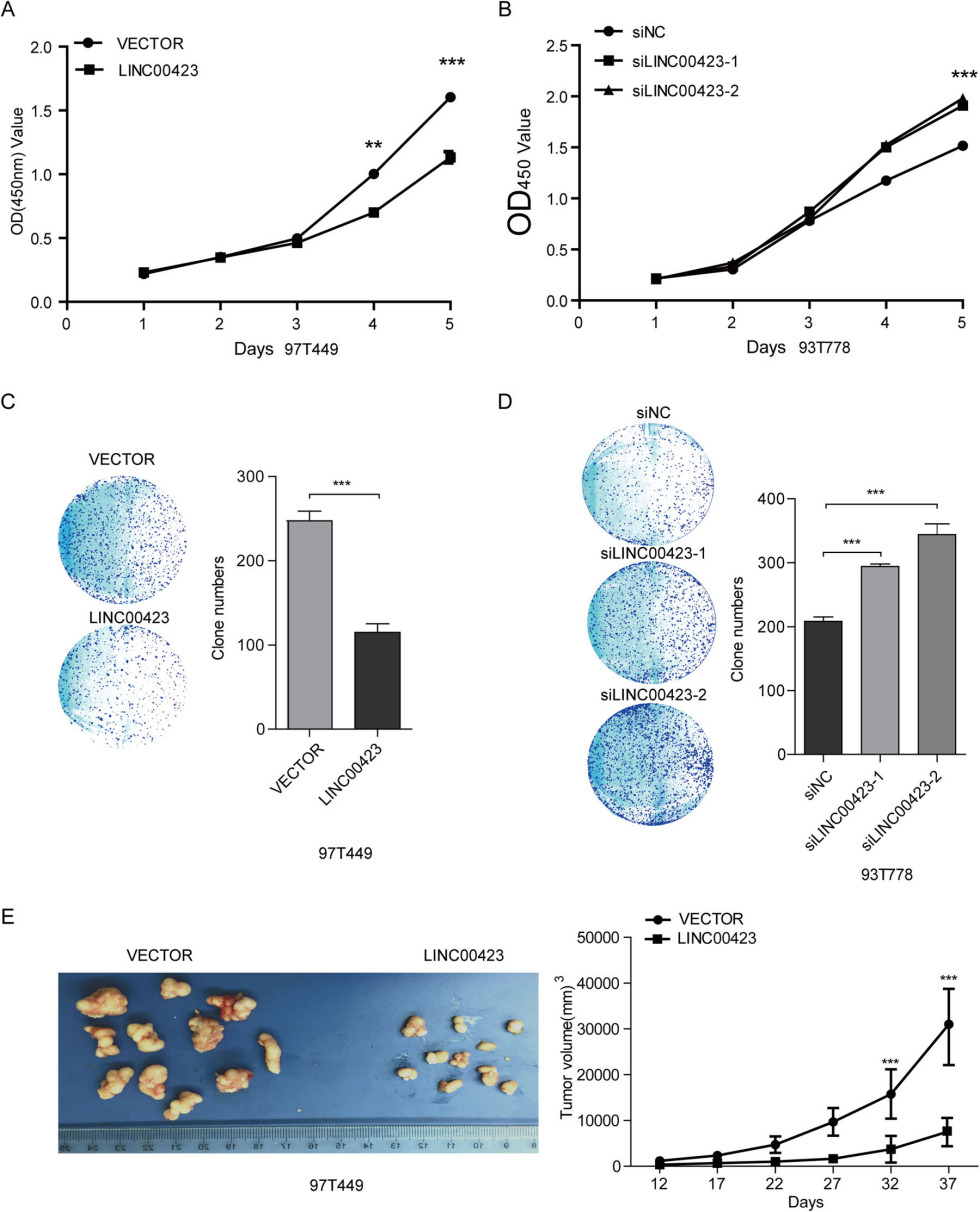
linc00423 inhibited RLS cell proliferation and colony formation in vitro and in vivo. **a** The CCK-8 assay to detected overexpressed of linc00423 inhibited the proliferation ability in 93T449 cells (***P < 0.001). **b** The CCK-8 assay to detected knockdown of linc00423 promoted the proliferation ability in 93T778 cells (***P < 0.001). **c** Overexpression of linc00423 decreased colony formation in 93T449 cells. The colonies was evaluated by crystal violet staining and counted (***P < 0.001). **d** Knockdown of linc00423 increased colony formation in 94T778 cells. (***P < 0.001). **e** The nude mouse xenograft model of linc00423 effect on proliferation of RLS cell lines. Lentiviral vector and linc00423-infected 93T449 cells (1 × 10^7^) were injected into the nude mouse. The tumor size and tumor volume were analyzed after 37 days after killing the nude mouse (***P < 0.001)

To assess the effect of linc00423 on RLS cell proliferation ability, we performed the CCK-8 and colony formation assay. We cloned full-sequence of linc00423 into the eukaryotic expression plasmid pcDNA3.0 to overexpressing linc00423 (Fig. 3g). The results showed that overexpression of linc00423 displayed a weakened viability in 97T449 cells (Fig. 4a). When we silenced linc00423 with two independent specific siRNAs, the proliferation was significantly increased for 93T778 cells (Fig. 4b). For the colony formation assay, we also confirmed that overexpression of linc00423 markedly decreased the proliferation, and knockdown of linc00423 accelerated the proliferation of 93T778 cells (Fig. 4c, d). We further explored the effect of linc00423 on RLS tumorigenesis in vivo. 97T449 cells stably transfected with pcDNA3.0-linc00423 or vector control were subcutaneously injected into nude mice, and the tumor was excised in 37 days. Tumor volume was smaller in the overexpressed linc00423 group than in the control group (*P* < 0.001). The tumor size followed the same pattern and was smaller in the overexpressed linc00423 group than in the control group (Fig. 4e). These investigations proved the tumor suppressor effect of linc00423 on RLS cells and such function might be attributed to its influence on cell proliferation by complex mechanisms.

### Linc00423 suppress RLS cell growth via binding with NFATC3

**Fig. 5:**
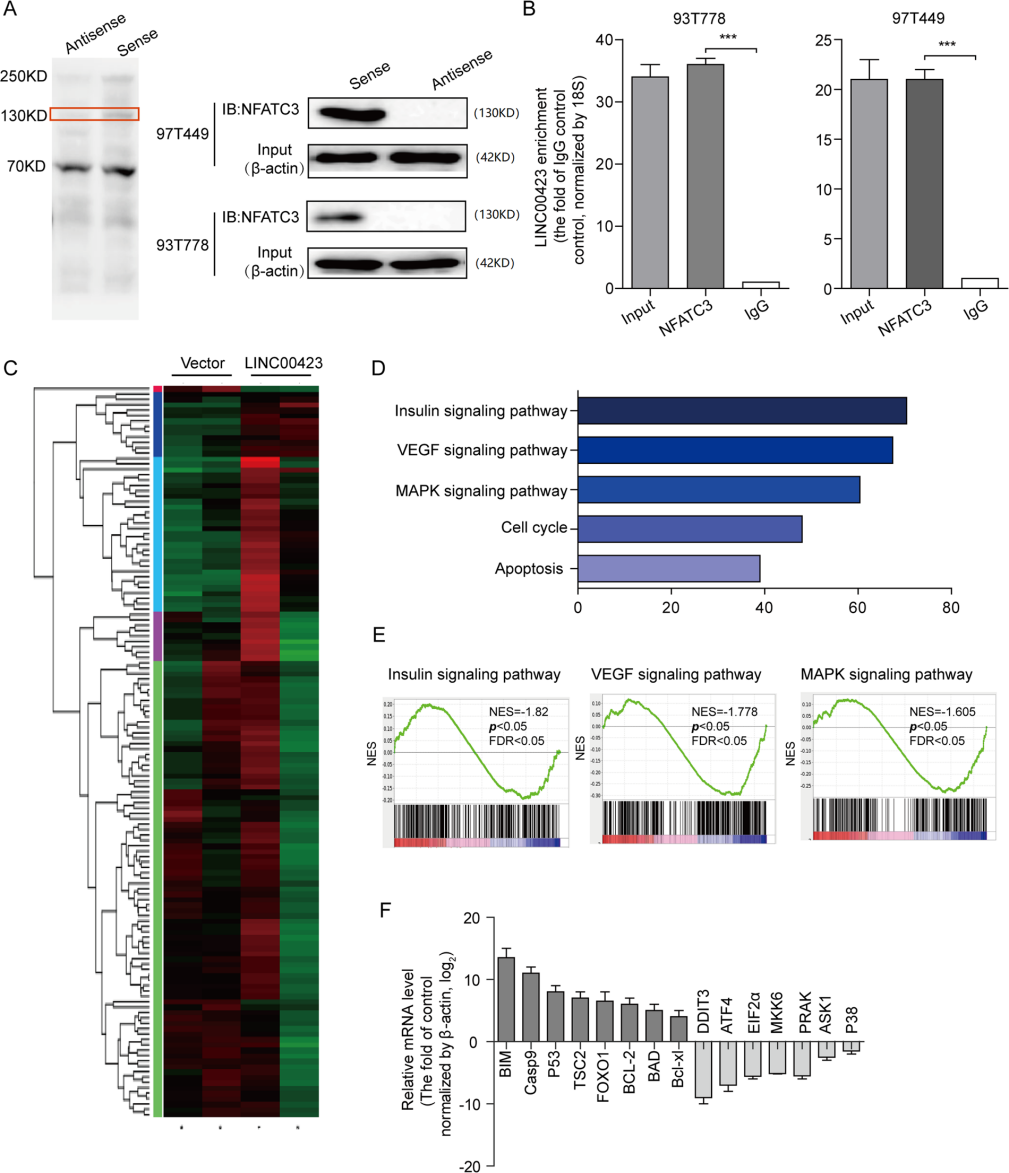
linc00423 involved in MAPK signaling pathway. **a** The RNA pulldown assay with silver staining showed linc00423-binding protein separated by SDS-PAGE in RLS cells. The protein bands were excised and detected by mass spectrometry analysis. Western-blot to analyze the interaction partners of linc00423 using RNA pulldown samples in two RLS cell lines. **b** RIP analyses were performed using antibodies against endogenous NFATC3, with IgG as a negative control. The RNA level of the linc00423 was detected using RT-PCR and normalized to the input. **c** Heat map showed clustering analysis of RNA sequencing by two independent vector and overexpressed linc00423 group. Each row represents a gene and each column represents a samples. Red represents upregulated genes and green represents downregulated genes. **d** GSEA analysis of RNA sequencing primary data when overexpressed linc00423. **e** Representative image of top three signaling pathways by GSEA analysis. **f** key genes of top three signaling pathways were verified by qRT-PCR when overexpressed linc00423 in RLS cells. **g** hybridization in situ and immunofluorescence exhibited the subcellular of linc00423 and NFATC3 in 97T449 cells

To search for protein partners of linc00423, the RNA pulldown assay was conducted with biotinylated linc00423, taken antisense linc00423 as a negative control and beads only as blank control. The eluent was analyzed by Mass spectrum. Excluding the nonspecific binding proteins, we identified only NFATC3 as a directly interacting protein of linc00423 by western blot of RNA pulldown samples in two cell lines (Fig. 5a). Furthermore, the RNA immunoprecipitation assay with an antibody against NFATC3 using cell extracts from the 93T778 and 97T449 cells also verified the interaction between NFATC3 and linc00423, and observed more linc00423 enrichment using the NFATC3 antibody than a nonspecific antibody (IgG control) in vitro (Fig. 5b).

To explore the molecular mechanisms, we performed RNA sequencing of overexpressed linc00423 and vector control cells to analyze the whole-genome profile affected by linc00423-mediated transcriptional regulation. In order to process the raw data, we used GSEA (Gene Set Enrichment Analysis) analysis, results showed top five signaling pathways and biological process, which include the insulin signaling pathway, VEGF signaling pathway, MAPK signaling pathway, cell cycle, and apoptosis (Fig. 5c–e). Based on the previous studies, the top three signaling pathways are coordinated to regulate the cell growth. Then, we selected 15 key regulator genes involved in these pathways to analyze whether these genes could respond to linc00423 overexpression. Results showed overexpressed linc00423 could increase the mRNA level of tumor suppressor genes such as casp9, p53, BCL-2, also decrease the mRNA level of oncogenes include DDIT3, EIF2α, MKK6, PRAK, and so on (Fig. 5f). These results suggest linc00423 as the key regulator involved in the MAPK signaling pathway, and the molecular mechanisms between them need to be further explored.

**Fig. 6:**
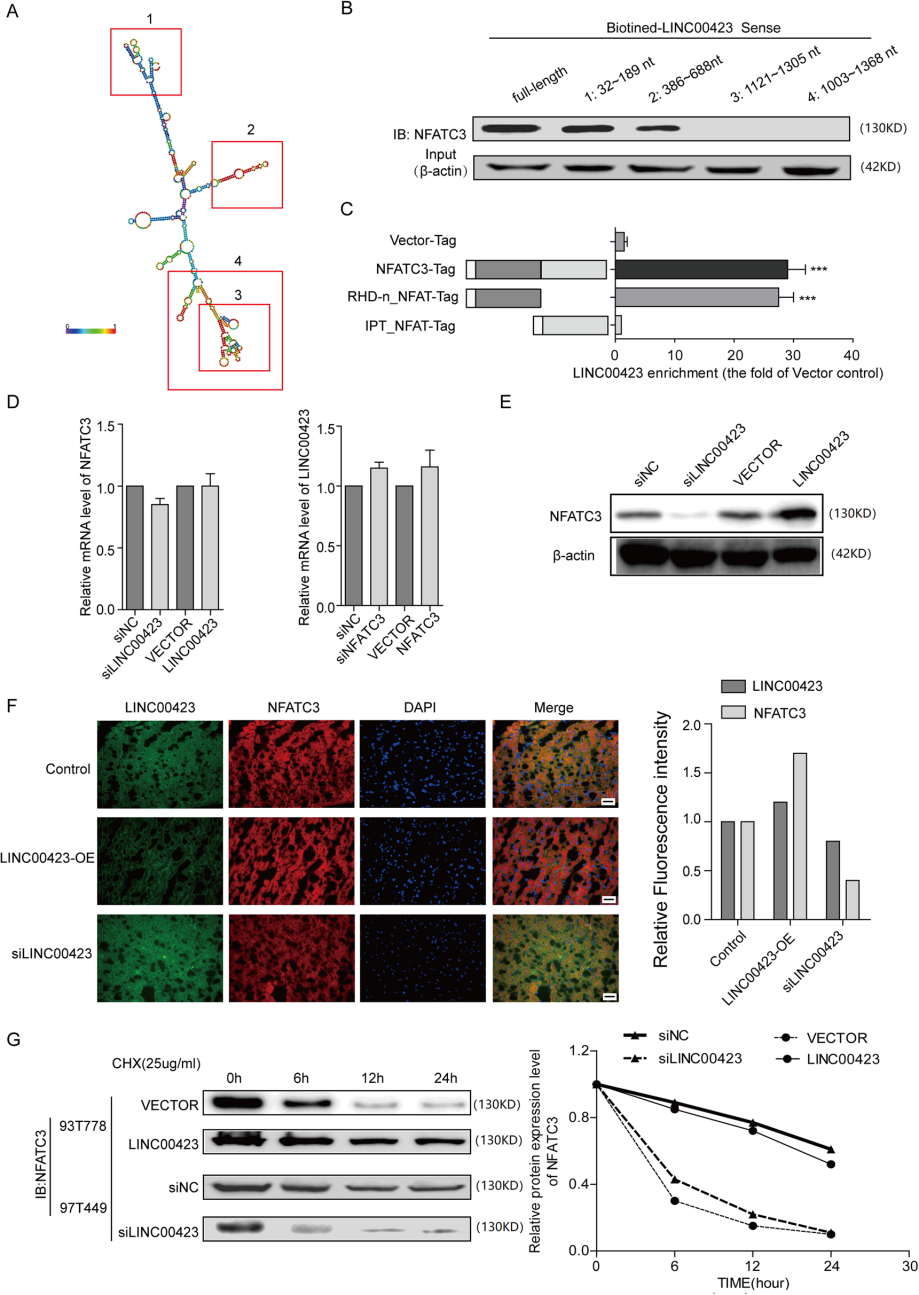
linc00423 specific directly binding with NFATC3 in RLS cells. **a** Deletion mapping of the linc00423 according to the second structure (https://lncipedia.org/). **b**, **c** The western blotting assay and qPCR assay detected the binding fragment/domains between linc0423 and NFATC3. **d** The qPCR assay detected the mRNA level of NFATC3 and RNA level of linc00423. **e**, **f** The western blotting assay and immunofluorescence assay detected the NFATC3 level under the treatment. **g** Overexpressed or knockdown of linc00423 were treated with protein synthesis inhibitor cycloheximide (CHX, 25 ug/ul) for 24 h. Detect the protein level of NFATC3 by western-blot

NFATC3 is a member of the nuclear factors of activated T-cells DNA-binding transcription complex^24,25^. Acts as a regulator of transcriptional activation in the MAPK signaling pathway, NFATC3 has played an important role in tumorigenesis^26,27^. In order to analyze the binding fragment/domain between linc00423 and NFATC3, we performed deletion mapping analyses and identified a 32–189-nt region and a 386–688-nt region at the 3′ end of linc00423 that is required for its association with NFATC3, and the RHD-n domain of NFATC3 was the vital domain mediate the interaction with linc00423 (Fig. 6a–c). We sought to determine the functional relevance of the association between linc00423 and NFATC3. Knockdown and overexpressed linc00423 has no effect on the mRNA level of NFATC3, and vice versa (Fig. 6d). Western blot and the immunofluorescence assay revealed knockdown of linc00423 could significantly reduce the protein level of NFATC3, and overexpressed linc00423 increased the expression level of NFATC3 (Fig. 6e, f). Treated with protein synthesis inhibitor CHX, we found linc00423 could regulated the NFATC3 protein stability. Results showed overexpressed linc00423 could prolong the half-time of NFATC3 in 93T778 cells, and when knockdown of linc00423 will accelerate the NFATC3 degradation (Fig. 6g). These findings suggest linc00423 by direct binding with and stabilization of NFATC3 to exert its tumor suppressor function in RLS.

### Linc00423 had a positive correlation with NFATC3 in RLS tissue

**Fig. 7:**
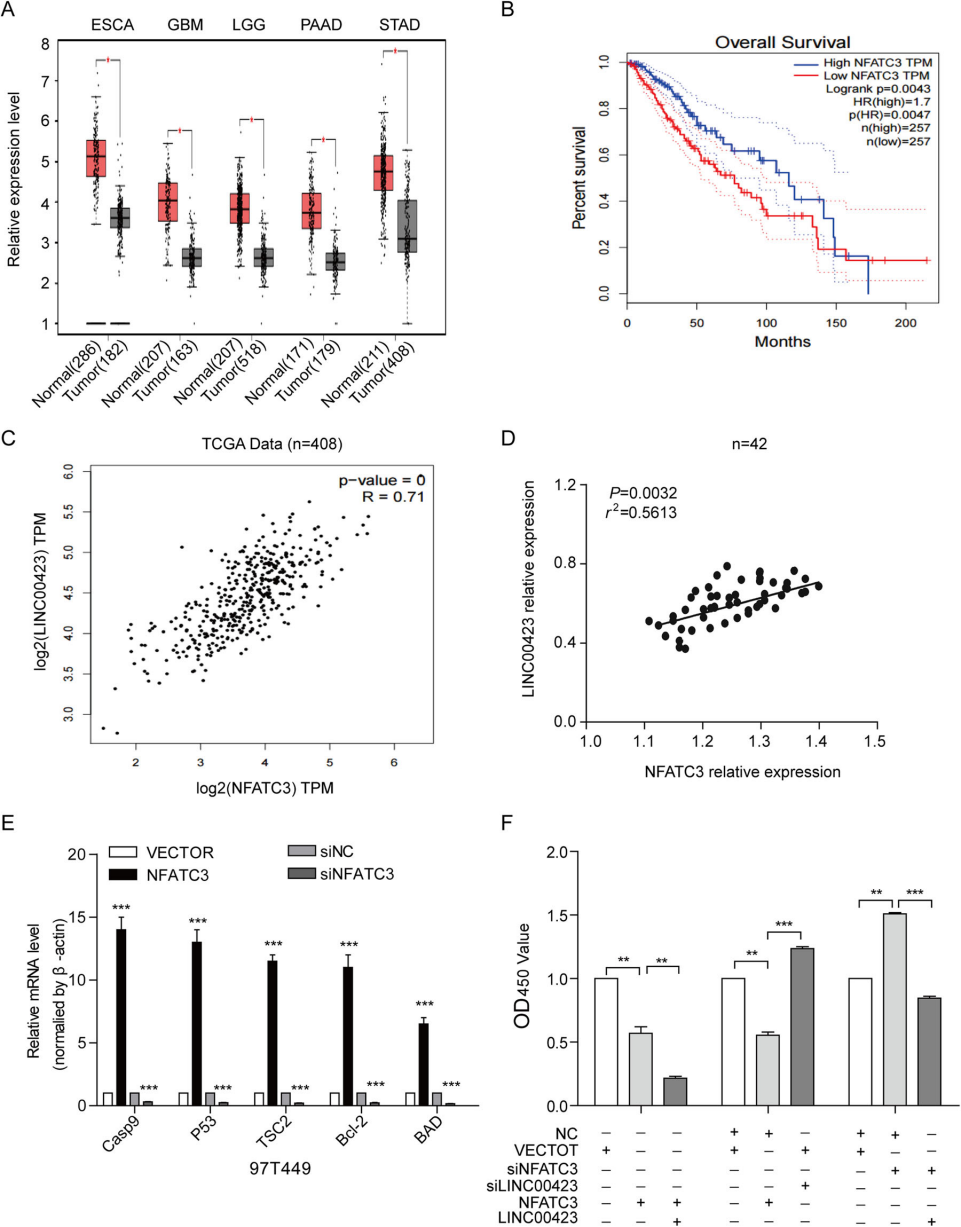
linc00423 suppress the cell proliferation via positive relation with NFATC3 in RLS tissues. **a** The expression panel of NFATC3 in various types of cancer. **b** Kaplan–Meier curves for prognistic value for NFATC3 in TCGA RLS cohort. **c**, **d** linc00423 had a positive relation with NFATC3 in TCGA cohort (**c**) and in 42 paired RLS tissues (**d**). **e** Key genes of MAPK signaling pathways were verified by qRT-PCR when overexpressed or knockdown of NFATC3 in RLS cells. **f** The recovery assay of NFATC3 in overexpression or knockdown linc00423 cells

NFATC3 had the significantly lower expression level in various types of cancers (Fig. 7a). And higher NFATC3 could predicted the better overall survival of RLS patients (Fig. 7b). To determine the correlation between linc00423 and NFATC3 in RLS, we first check the correlation in 408 paired TCGA data, result showed they have a positive correlation in RLS tissue (Fig. 7c). Besides, we also confirmed the results in 42 paired of RLS tissue (Fig. 7d). We also evaluate the properties and effects of NFATC3 in RLS. We established NFATC3 stable overexpression RLS cell lines and effective siRNAs (Fig. S2A). Results showed knockdown of NFATC3 leads to increase the colony information and the size of colonies. And overexpression of NFATC3 expression in 97T449 cells leads to a significant decrease of proliferation (Fig. S2B, C), and vice versa. Besides, alteration of NFATC3 could contribute the malignancies of RLS cells (Fig. S2D). These results revealed NFATC3 as a tumor suppressor in RLS cancer cells.

On our study, NFATC3 as the downstream effect target genes of linc00423. These finding also verified the molecular mechanisms between them. In addition, we found that overexpression of NFATC3 upregulated Casp9, p53, TSC2, Bcl-2, and BAD, in turn, knockdown of NFATC3 reduce the expression level of these genes (Fig. 7e). From above results, we safely conclude that both linc00423 and NFATC3 play a negative role in tumorigenesis. At last, we performed the cell function recovery assay. Results showed overexpressed NFATC3 could decreased the cell proliferation and otherwise knockdown NFATC3 could increase the proliferation ability. Results in the functional recovery assay also demonstrated when cooverexpressed linc00423 and NFATC3 in RLS cells, could significantly inhibited proliferation ability contrast with overexpressed NFATC3 only. When overexpressed linc00423 in knockdown NFATC3 cells will inhibited the cell proliferation contrast with knockdown NFATC3 only (Fig. 7f). Taken together, both linc00423 and NFATC3 were as tumor suppressors in RLS, they make a meaningful contribution to cell proliferation through regulated the MAPK signaling pathway.

## Discussion

Mounting lncRNAs have been shown to be associated with cancer development and progression, demonstrating potential applications as novel diagnostic or prognostic molecular markers in clinical management and treatment^13,28,29^. RLS is the most common STS, due to the lack of characteristic clinical presentations, both preoperative diagnosis and prognosis of patients face severe challenges^30–32^. Fully understanding the molecular mechanism of RLS development and progression have a great significance for treatment of RLS patients.

In this study, we identified a novel lncRNA-linc00423 involved in RLS tumorigenesis and progression by binding with NFATC3. The nuclear factor of activated T-cells (NFAT) family comprises five calcium-dependent transcription factors, in which NFATC3 is a predominant NFAT gene that has been implicated in the pathogenesis of various inflammatory pathologies. NFATC3-deficiency alleviated inflammatory response and apoptosis through inactivating NF-kB (p65), Caspase-3 and MAPKs (p38 and JNK) signaling pathways. As reported, NFATC3 partly has a parallel function as NF-kB to promote inflammatory response which also expressed and is the target of immune receptor signals, lead to a rapid rise of intracellular Ca^+^, to activation of cytosolic NFAT proteins^26,33–35^. But until now, the biological functions of NFATC3 were still undefined. Through our study on the mechanisms driven by linc00423, found knockdown of LINC00423 has closely related with MAPK signaling pathways and linc00423 could directly binds to NFATC3 and increase its protein stability. Therefore, linc00423 could increase the protein stability of NFATC3 which could blocked p38 and JNK activation. Recent study shows that MAPKs' activation is a major cause that contributes to tumor progression. In our study, we, for the first time, demonstrated that NFATC3 can bind with lncRNA, which is regulated by linc00423, and NFATC3 as the tumor suppressor in RLS. The present study revealed that NFATc3 might be a key modulator and plays an essential function under pathological conditions.

To sum up, linc00423 had a significantly downregulated in RLS tissues, and overexpression of linc00423 inhibited RLS cell growth through the negatively regulated MAPK signaling pathway. For the first time we demonstrated NFATC3 can bind with lncRNA, and NFATC3 as the tumor suppressor in RLS. Linc00423 negatively regulated the MAPK signaling pathway, which participated in regulating RLS cell carcinogenesis. Therefore, our study implies that the linc00423 may provide a potential new target for RLS treatment.

## Supplementary information

Supplementary information
