# Supplementary material for: Linc00423 as a tumor suppressor in retroperitoneal liposarcoma via activing MAPK signaling pathway through destabilizing of NFATC3
Source: Cell Death Dis. 2019 Jun 3;10(6):430. doi: 10.1038/s41419-019-1658-2 (PMC6546787; doi:10.1038/s41419-019-1658-2)
Supplement: Supplementary file 1 — Supplementary information [file 41419_2019_1658_MOESM1_ESM.docx]

**Linc00423 as a Tumor Suppressor in Retroperitoneal Liposarcoma via activing MAPK signaling pathway through** **destabilizing of NFATC3**

Yong Zhang^1^*, Hanxing Tong^1^*, Junyi He^1^*, Yebo Shao^1,2^*, Xi Guo^3^, Rongyuan Zhuang^3^, Jue Yang^4^, Ju Liu^5^, Yuqin Ding^6^, Wenshuai Liu^1^, Weiqi Lu^1#^,Yuhong Zhou^2#^ .

^1^Department of General Surgery, Zhongshan Hospital, Fudan University, Shanghai, 200032, China.

^2^Department of Oncology, Zhongshan Hospital, Fudan University, Shanghai, 200032, China.

^3^Department of General Surgery, Xiamen Branch, Zhongshan Hospital, Fudan University, Xiamen, 361015, China

^4^Department of Vascular Surgery, Zhongshan Hospital, Fudan University, Shanghai, 200032, China.

^5^Department of Pathology, Zhongshan Hospital, Fudan University, Shanghai 200032, China

^6^Department of Radiology, Zhongshan Hospital, Fudan University, Shanghai, 200032, China.

*These authors contributed equally to this work

**Short title:** Linc00423 plays the tumor-suppressor in retroperitoneal liposarcoma.

^#^Co-corresponding authors.

Weiqi Lu, Department of General Surgery, Zhongshan Hospital, Fudan University, 180 Fenglin Road, Shanghai 200032, China . Tel/Fax: 021-64041990; Email: [lu.weiqi@zs-hospital.sh.cn](mailto:lu.weiqi@zs-hospital.sh.cn); Yuhong Zhou, Department of Oncology, Zhongshan Hospital, Fudan University, 180 Fenglin Road, Shanghai 200032, China. Tel/Fax: 021-64041990; Email: [yhzhouzsht@163.com](mailto:yhzhouzsht@163.com)


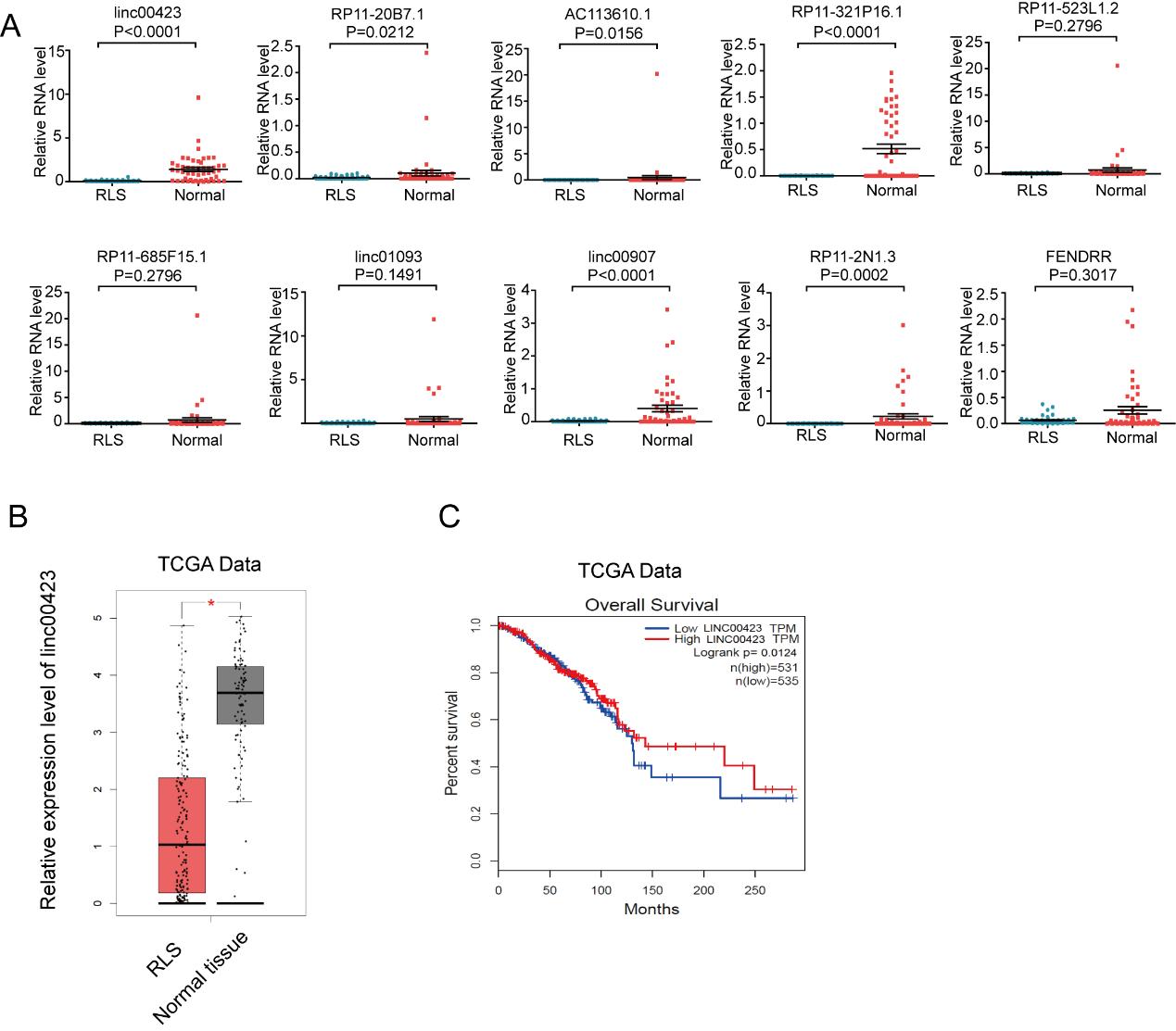


Supplementary Figure 1

A: RNA level of ten candidate long non-coding RNA from the RNA-seq data.

B: The expression profile of linc00423 in 408 paired scrcoma and paired normal tissues of TCGA data.

C: Kaplan-Meier curves for prognistic value for linc00423 in TCGA RLS corhort.


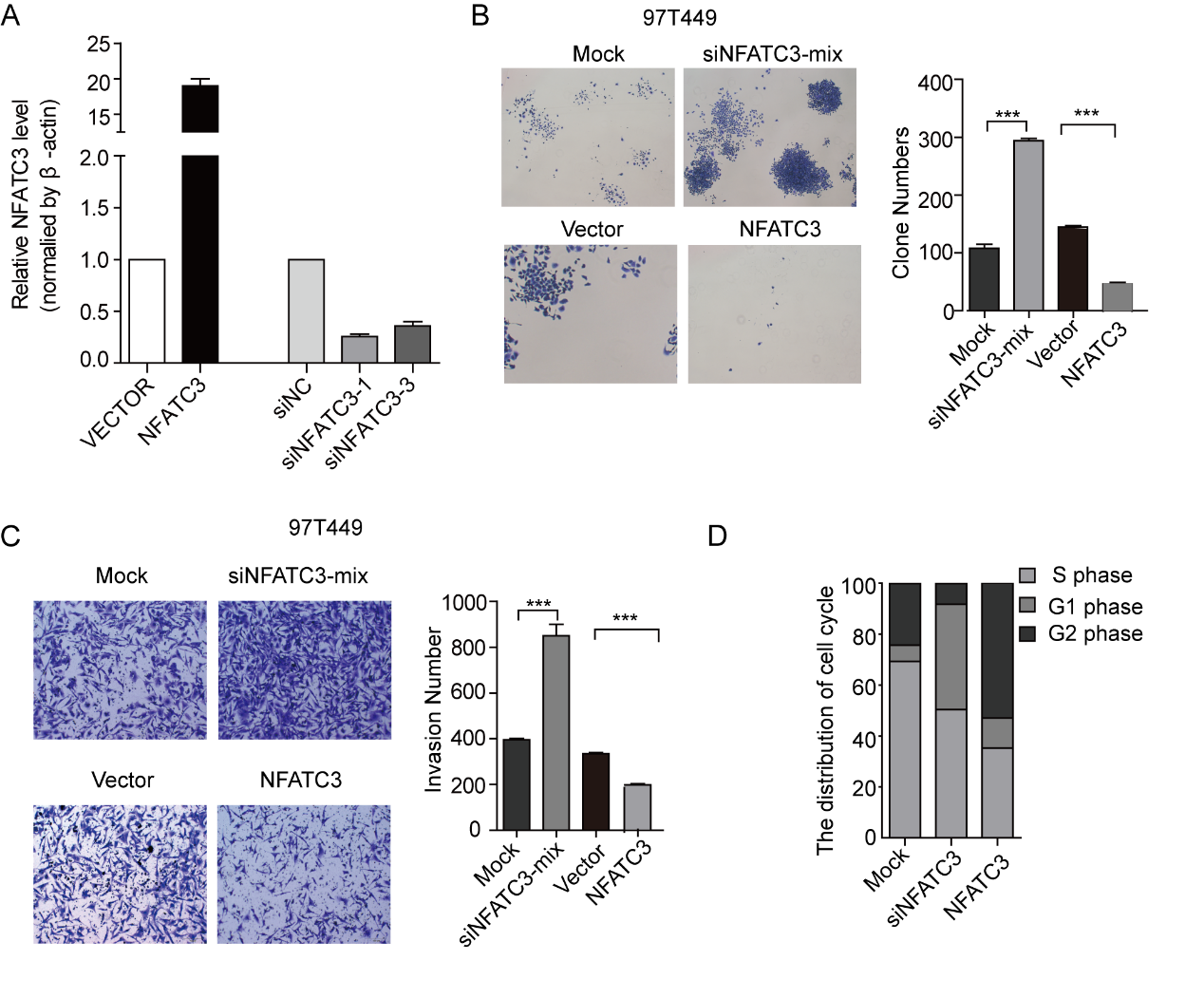


Supplementary Figure 2: NFATC3 as the tumor-suppressor in RLS cells

A: The efficiency of overexpression and knockdown of NFATC3 in RLS cell lines.

B, C: Knockdown of NFATC3 enhanced the RLS cell colony formation and invasion;

NFATC3 overexpression represses the proliferation and invasion of RLS cell. The invasion number of cells was evaluated by crystal violet staining and counted (***: P<0.001).

D: The alteration of cell cycle distribution when knockdown or overexpressed NFATC3.
